# Supplementary material for: The Rice Alpha-Amylase, Conserved Regulator of Seed Maturation and Germination
Source: Int J Mol Sci. 2019 Jan 21;20(2):450. doi: 10.3390/ijms20020450 (PMC6359163; doi:10.3390/ijms20020450)
Supplement: Supplementary file 1 [file ijms-20-00450-s001.pdf]

The Rice Alpha-Amylase, Conserved Regulator of Seed Maturation and Germination

Supplementary Figures

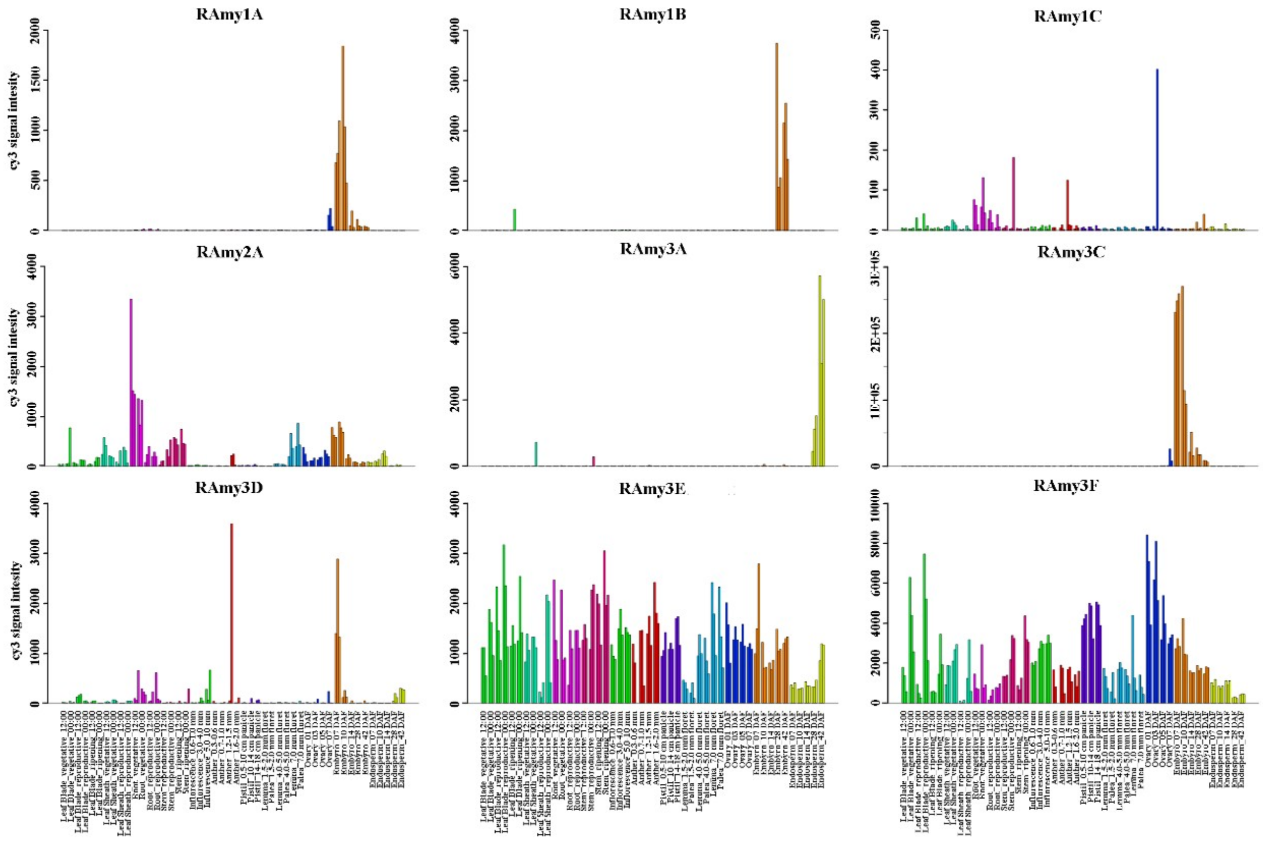

Supplementary figure 1. Expression of rice alpha-amylase isozymes during the entire life cycle of rice, exhibiting expression differences in various tissues. RAmy1A, RAmy1B, RAmy3A and RAmy3C were highly expressed during seed development and maturation while RAmy1C, RAmy2A, RAmy3D, RAmy3E and RAmy3F were expressed during the entire growth period. The figures were downloaded from the http://ricexpro.dna.affrc.go.jp/ website.

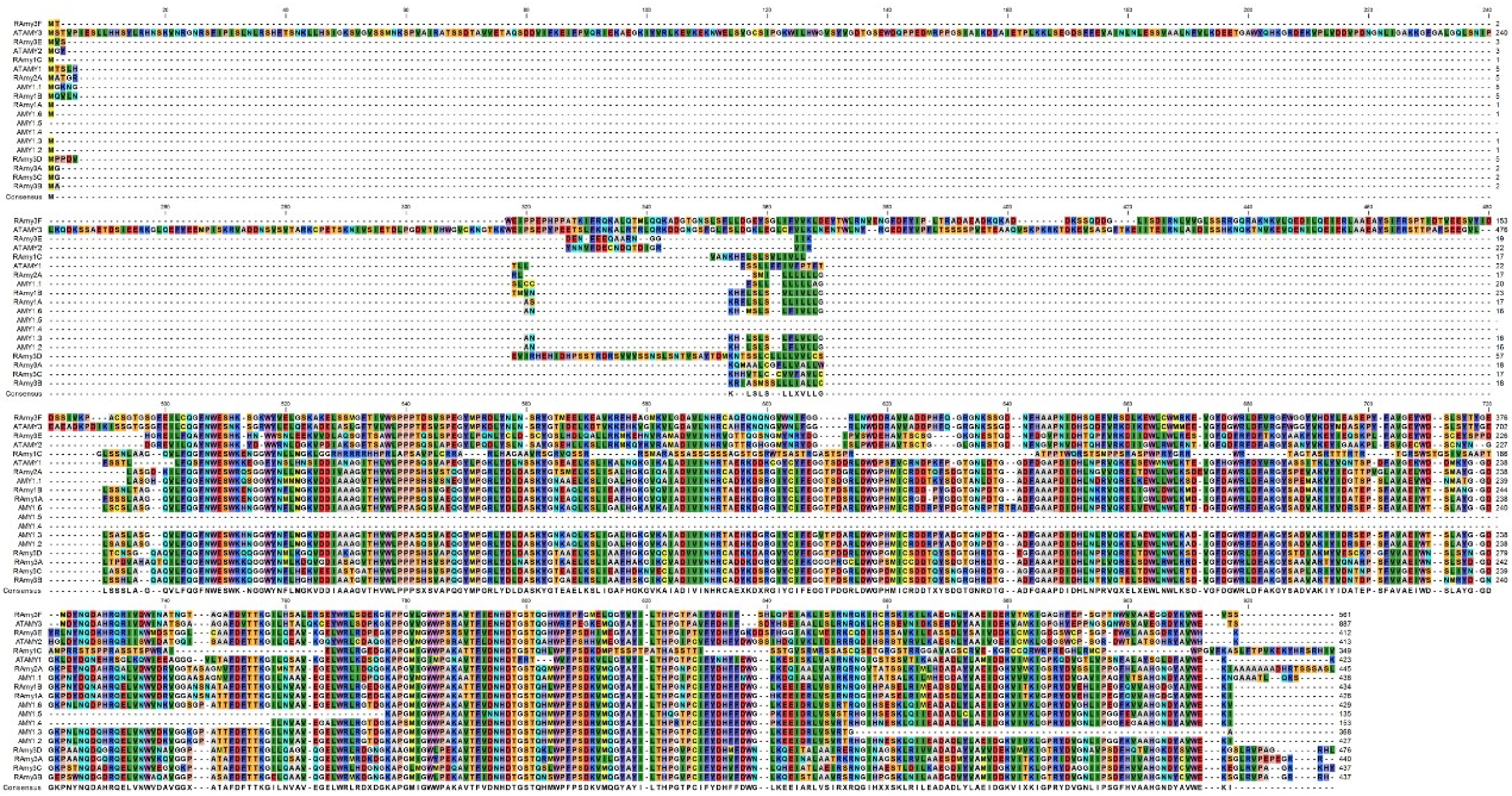

Supplementary Figure 2. Sequence alignment for rice, barley and *Arabidopsis* alpha-amylase protein. The protein sequences were obtained from the Rice Genome Annotation Project using LOC_Os02g52700, LOC_Os02g52710, LOC_Os01g25510, LOC_Os06g49970, LOC_Os09g28400, LOC_Os09g28420, LOC_Os08g36900, LOC_Os08g36910, LOC_Os04g33040, and LOC_Os01g51754 for *RAmy1A*, *RAmy1B*, *RAmy1C*, *RAmy2A*, *RAmy3A*, *RAmy3B*, *RAmy3C*, *RAmy3D*, *RAmy3E*, and *RAmy3F*, respectively. The barley and Arabidopsis sequences were downloaded from NCBI and TAIR respectively.
